# Latest Insights into Unique Open Reading Frames Encoded by Unique Long (UL) and Short (US) Regions of Marek’s Disease Virus

**DOI:** 10.3390/v13060974

**Published:** 2021-05-25

**Authors:** Yifei Liao, Blanca Lupiani, Sanjay M. Reddy

**Affiliations:** Department of Veterinary Pathobiology, College of Veterinary Medicine & Biomedical Sciences, Texas A&M University, College Station, TX 77840, USA; liao.yifei@tamu.edu (Y.L.); blupiani@tamu.edu (B.L.)

**Keywords:** Marek’s disease virus, genome, open reading frame, replication, pathogenesis

## Abstract

Marek’s disease virus (MDV) is an oncogenic avian alphaherpesvirus whose genome consists of unique long (UL) and short (US) regions that are flanked by inverted repeat regions. More than 100 open reading frames (ORFs) have been annotated in the MDV genome, and are involved in various aspects of MDV biology and pathogenesis. Within UL and US regions of MDV, there are several unique ORFs, some of which have recently been shown to be important for MDV replication and pathogenesis. In this review, we will summarize the current knowledge on these ORFs and compare their location in different MDV strains.

## 1. Introduction

Marek’s disease (MD) is an infectious and neoplastic disease of chickens caused by an oncogenic alphaherpesvirus, *Gallid alphaherpesvirus* 2 (GaHV-2), also known as Marek’s disease virus (MDV). Infection with MDV causes neurological disease, immunosuppression, paralysis, blindness, and lymphomas in chickens. MDV was originally thought to be a gammaherpesvirus due to its ability to induce rapid onset of T-cell lymphoma in chickens. In the early 1980s, an electron microscopy study revealed that the genome of MDV and its close relative, *Meleagrid alphaherpesvirus* 1 (MeHV-1) or turkey herpesvirus (HVT), consists of two unique regions, each flanked by inverted repeat regions [1], indicating that MDV is genetically related to alphaherpesvirus. This observation was further confirmed by whole genome sequencing [2,3,4]. According to current taxonomy, along with its close relatives GaHV-3 (also known as MDV-2) and HVT, MDV is classified as a member of *Mardivirus* genus in the *Alphaherpesvirinae* subfamily, family *Herpesviridae*. In addition, MDV strains vary in their virulence and are classified into different pathotypes, including mild (m), virulent (v), very virulent (vv), and very virulent plus (vv+). It is generally accepted that infection of MDV causes four phases of pathogenesis in susceptible chickens, including early cytolytic phase, latent phase, late cytolytic/immunosuppressive phase, and proliferative phase [5]. Feather follicle epithelium (FFE) is the only place where fully infectious stable MDV particles are produced, allowing for horizontal transmission via infectious dander shed from chickens [5].

In 1969, a serial-passage attenuated MDV, HPRS-16/att, was first used as a vaccine to control MD [6]. Later, a mildly oncogenic MDV strain, CVI988/Rispens, was isolated and attenuated by serial cell culture passage, which provided superior protection than HPRS-16/att did [7,8,9]. In addition, HVT and MDV-2, which do not cause disease in chickens, have also been used individually or in combination to control MD [10,11,12]. Although efficient in controlling MD, MDV vaccines do not provide sterilizing immunity against field strains. Thus, it is generally believed that the use of vaccines contributed to the evolution of MDV field viruses towards increased virulence. Because of superior protection against highly virulent MDV, CVI988/Rispens is currently considered the “gold standard” of MD vaccines. In the last two decades, development of herpesvirus genome manipulation methods has allowed researchers to identify and study pathogenesis and tumorigenesis- related genes and generate recombinant MDV vaccine candidates, such as vector vaccines, DNA vaccines, and gene deletion vaccines [13].

In 2000, genomes of GA (GenBank: AF147806.2) and Md5 (GenBank: AF243438.1) strains of MDV were fully sequenced and more than one hundred open reading frames (ORFs) were identified and annotated [3,4], advancing studies focused on MDV molecular biology, pathogenesis, and vaccine development to a new era. The MDV genome consists of unique long (UL) and short (US) regions, each flanked by inverted terminal and internal repeat long (TRL, IRL) and short (TRS, IRS) regions, respectively. Even though the genes are highly conserved, the nomenclatures of genes in GA and Md5 strains are different. In the GA strain, MDV genes were named after herpes simplex virus 1 (HSV-1) homologues; while the MDV-specific ORFs were named LORFs, R-LORFs, SORFs, or R-SORFs, based on the location of the start codon within the UL, RL, US, or RS region, respectively [3]. However, Md5 genes were simply named contiguously (MDV001, MDV002…) in the order of TRL-UL-IRL-IRS-US-TRS [4].

When compared to other alphaherpesviruses, genes such as *meq*, *vIL8*, and *vTR*, encoded within the repeat regions, are specific to MDV and have been shown to be important for MDV pathogenesis and/or oncogenesis, and have been summarized previously [14,15,16]. In addition, by deleting the entire internal repeat regions (IRL and IRS), a recent study showed that both copies of inverted repeat regions of MDV are required for efficient virus replication and pathogenesis in vivo but dispensable for virus replication and cell-to-cell spread in vitro [17]. Although most genes encoded by the UL and US regions of MDV are thought to be functional homologues of HSV-1, there are some ORFs that are unique to MDV. Due to the presence of two different nomenclatures, the presence and location of these ORFs are confusing. In addition, some of these ORFs have been characterized in the past 20 years. Thus, in this review, we aim to compare the location of these ORFs in the genome of different MDV strains, and summarize current knowledge towards these ORFs.

## 2. MDV UL Unique ORFs

**Figure 1:**
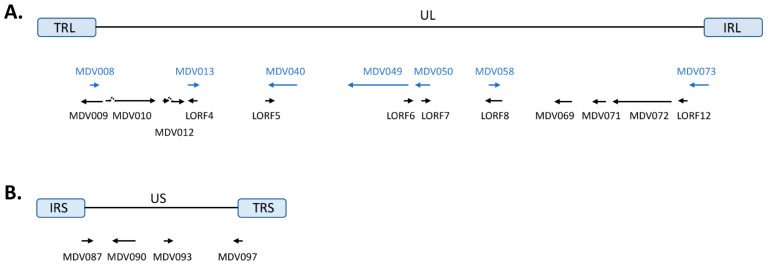
Genome structure and location of MDV unique open reading frames (ORFs). (**A**) MDV unique ORFs in the UL region of the Md5 strain are presented. Overlapping ORFs, displayed in blue, are also presented. (**B**) MDV unique ORFs in the US region of Md5 strain are presented.

Proteins encoded by genes in the UL region of MDV are involved in various aspects of MDV biology, such as DNA replication, gene expression, virion packaging, and morphogenesis. In the UL region of Md5 strain, a total of 12 unique ORFs were identified (Figure 1A), and the nucleotide location of each ORF is listed in Table 1. In addition, we analyzed the presence of their homologues in genomes of representative strains from different MDV pathotypes, including v MDV (GA), vv MDV (RB-1B), vv+ MDV (648a), and vaccine strain CVI988, and provided the nucleotide location information in Table 1. In cases where an ORF is present but has not been annotated, we analyzed the genome sequences of these MDV strains and provided the predicted nucleotide location (Table 1). In the last two decades, functional characterization of these ORFs uncovered some important functions.

### 2.1. MDV009/LORF1

MDV009/LORF1 (Md5, nucleotide: 14,338–13,337) is located at the TRL/UL junction region of Md5 genome, and encodes a 333 amino acids (aa) long protein, with a predicted molecular weight (MW) of 37.9 kDa. It is in opposite orientation to MDV008/R-LORF14 (Md5, nucleotide: 13,833–14,300) which encodes phosphoprotein pp24. MDV009/LORF1 is unique to MDV-1 and has no homologue in MDV-2, HVT, and other non-avian herpesviruses [4]. Even though expression of MDV009/LORF1 mRNA was detected in an MDV- transformed tumor cell line [18], its function in MDV pathogenesis remains to be studied.

### 2.2. MDV010/LORF2

MDV010/LORF2 (Md5, nucleotide: 14,535–14,630, 14,701–16,875) is located at the left terminus of the UL region (where UL1 is located), and is a spliced gene that encodes a 756-aa long viral lipase (vLIP) protein. MDV010/LORF2 homologues are present in MDV-1, MDV-2, and HVT [4,19]. Lipases are a group of enzymes that catalyze the hydrolysis of lipids and are widely present in a variety of hosts, including plants, animals, and prokaryotes, and have been shown to play important roles in cell metabolism, immunity, and signal transduction [20,21,22]. Some DNA viruses encode proteins equipped with lipase activities, such as vaccinia virus major envelope protein p37 which exhibits broad lipid-metabolizing activities, and VP1 capsid proteins of parvovirus and adeno-associated virus type 2 (AAV-2) which contain a phospholipase A2 (PLA2) motif that is critical for virus infectivity [23,24,25].

MDV vLIP is the first reported herpesvirus lipase and aa 229–369 shows significant homology to the critical structure of pancreatic lipase α/β hydrolase fold, a protein-folding structure that is important for the enzymatic activity of pancreatic lipase [3,26,27]. vLIP has been suggested to be dispensable for MDV replication in vitro since insertion of a long terminal repeat (LTR) from reticuloendotheliosis virus (REV) into the MDV010/LORF2 nucleotide sequence of JM strain resulted in a new strain, JM-Hi3, which was not impaired for in vitro replication [3,28]. On the other hand, Kamil et al. showed that vLIP is not required for MDV growth in vitro, but it is important for MDV replication and pathogenesis in vivo as both deletion of the entire vLIP coding sequence and mutation of a serine nucleophile position caused reduced lytic replication of MDV, lower tumor incidence, and higher survival rate in chickens [26]. This research team also characterized functions of MDV vLIP, and found that MDV010/LORF2 is a late gene, and vLIP is a secreted glycoprotein lacking detectable lipase activity, suggesting it may be an unconventional lipase [26].

### 2.3. MDV012/LORF3

In a more recent Md5 genome annotation (GenBank: NC_002229.3), two originally annotated ORFs MDV011* (Md5, nucleotide: 17,431–17,688) and MDV012* (Md5, nucleotide: 17,828–18,982) were re-annotated as a single spliced ORF, named MDV012 (Md5, nucleotide: 17,431–17,539, 17,622–18,982), which has been shown to encode protein p012 [29]. However, in the corresponding region of GA, only one gene, LORF3 (GA, nucleotide: 16,351–17,547), was annotated and is 42 nucleotides longer than MDV012*. Based on our sequence analysis results, an ORF (GA, nucleotide: 15,987–16,245) corresponding to MDV011* is present in the GA genome (Table 1), and can potentially splice with LORF3 as shown for Md5. On the other hand, even though only LORF3 (648a, nucleotide: 17,247–18,401) was annotated in 648a, it has same size as MDV012* of Md5 (Table 1). In addition, a homologue (648a, nucleotide: 16,850–17,107) of Md5 MDV011* is present in the 648a genome (Table 1). MDV012 homologues are also present in MDV-2, HVT, falconid herpesvirus (FaHV), infectious laryngotracheitis virus (ILTV), and duck enteritis virus (DEV) [29,30], suggesting that it plays an important role in avian alphaherpesvirus biology.

Schippers et al. studied the importance of MDV012 in regulating MDV replication and characterized the properties of p012 protein [29]. They found that an MDV012 deletion mutant exhibited severely impaired in vitro growth properties and failed to be passaged in cell culture, suggesting that MDV012 is essential for virus growth in vitro [29]. They also found that p012 is primarily located in nuclei of infected and transfected cells, and the nuclear export of p012 could be blocked by leptomycin B, a potent protein nuclear export inhibitor, suggesting that p012 could shift between nucleus and cytoplasm [29]. Further, a functional nuclear localization signal was mapped to the C-terminus of p012, which could transfer a fused green fluorescent protein (GFP) to nucleus [29]. In addition, p012 appeared to be phosphorylated as treatment with a protein phosphatase resulted in reduced molecular weight in sodium dodecyl sulfate polyacrylamide gel electrophoresis (SDS-PAGE) and Western blotting [29]. Later, the MDV012 gene product was shown to be capable of reducing the expression of major histocompatibility complex (MHC) class I on the cell surface, which could be partially blocked by ectopic expression of MHC class I-binding peptide [30]. This study suggests that MDV012, like UL49.5 protein (encoded by MDV064), is a novel MHC class I immune evasion gene.

### 2.4. LORF4

LORF4 (Md5, nucleotide: 19,608–19,180) encodes a 142-aa long protein with a predicted MW of 16 kDa. It is antisense to MDV013/UL1 (Md5, nucleotide: 19,172–19,759) which encodes glycoprotein L (gL). LORF4 is unique to MDV-1 and is not present in MDV-2 and HVT. Using a two-hybrid screening method and in vitro binding assays, Niikura et al. identified an interaction between the protein encoded by LORF4 and chicken MHC class II beta chain [31]. Later, they generated a mutant Md5 where the start codon of LORF4 was disrupted and a premature stop codon was introduced without affecting the amino acid sequence of gL [32]. They found that LORF4 is dispensable for virus growth in vitro; however, disruption of LORF4 appears to increase pathogenicity of MDV [32]. As of now, the significance of LORF4 and chicken MHC class II interplay remains to be studied.

### 2.5. LORF5

LORF5 (Md5, nucleotide: 60,996–61,355) encodes a 119-aa long protein with a predicted MW of 13.1 kDa. A total of 295 nucleotides at the 3′ end of LORF5 overlap with MDV040/UL27 (Md5, nucleotide: 63,652–61,055), which encodes gB, a major membrane glycoprotein. LORF5 is unique to MDV-1 and is not present in MDV-2 and HVT genomes. In 2000, Schumacher et al. characterized a gB mutant MDV where 2 kb nucleotides of gB, including 49 nucleotides at 3′ end of LORF5, were deleted [33]. Their study showed that this deletion impaired cell-to-cell spread of the mutant virus, which could be restored in gB-expressing cells, suggesting that gB, and not the 49 nucleotides at 3′ end of LORF5, is essential for virus spread in vitro [33].

### 2.6. LORF6

LORF6 (Md5, nucleotide: 88,119–88,586) encodes a 155-aa long protein with a predicted MW of 17.2 kDa. It is antisense to the 5′ end of MDV049/UL36 (Md5, nucleotide: 88,471–78,443) which encodes UL36 major tegument protein. LORF6 is unique to MDV-1 and is not present in MDV-2 and HVT. The importance of LORF6 in MDV pathogenesis remains unknown.

### 2.7. LORF7

LORF7 (Md5, nucleotide: 91,537–91,899) encodes a 120-aa long protein with a predicted MW of 12.9 kDa. It is antisense to 5′ end of MDV050/UL37 (Md5, nucleotide: 91,826–88,686) which encodes UL37 tegument protein. LORF7 is unique to MDV-1 and is not present in MDV-2 and HVT genomes. The importance of LORF7 in MDV pathogenesis remains unknown.

### 2.8. LORF8

LORF8 (Md5, nucleotide: 104,992–104,366) encodes a 208-aa long protein with a predicted MW of 23.0 kDa. It is antisense to MDV058/UL45 (Md5, nucleotide: 104,532–105,167), which encodes UL45 envelope transmembrane protein. LORF8 is unique to MDV-1 and is not present in MDV-2 and HVT. Both LORF8 and MDV058/UL45 are located downstream of MDV057/UL44, which encodes gC. Previously, Tischer et al. showed that both deletion of the entire gC coding region and mutation of the start codon of gC resulted in larger MDV plaque size; in addition, plaque size of the gC deletion virus was larger than that of the gC start codon mutant virus, suggesting that plaque size differences might be due to downstream LORF8 or MDV058/UL45 genes, since deletion of the entire gC coding sequence may affect their expression [34].

### 2.9. MDV069/LORF9

MDV069/LORF9 (Md5, nucleotide: 121,289–120,480) encodes a 269-aa long protein with a predicted MW of 29.7 kDa, and is located at the right terminus of the UL region. MDV069/LORF9 homologues are present in MDV-1, MDV-2, and HVT [4,19]. MDV069/LORF9 has been shown to be important for MDV early cytolytic replication and pathogenesis [35,36]. We have reported that deletion of LORF9 from a vv+ MDV, 686 strain, did not affect virus growth in cell culture, while replication of the LORF9 deletion virus in lymphoid organs, including spleen, thymus, and bursa, was significantly impaired during the early cytolytic phase [36]. Similarly, replication of LORF9 deletion virus in the feather follicle epithelium (FFE) was also partially impaired when compared to parental virus [36]. This study also showed that deletion of LORF9 attenuated MDV pathogenicity causes 30.8% mortality and 84.6% tumor incidence, compared to 100% mortality and tumor incidence caused by parental virus [36].

Meq is the major oncogene of MDV, which is essential for MDV tumorigenicity [37]. Mutant virus with both copies of *meq* deleted did not cause tumors in infected chickens and provided superior protection than CVI988/Rispens does against MD caused by highly virulent MDV [37,38,39]. However, a *meq* deletion virus retains the ability to cause lymphoid organ atrophy in chickens [40]. By deleting a second gene, LORF9, Sun et al. showed that deletion of LORF9 could eliminate the lymphoid organ atrophy induced by a *meq* deletion virus [35]. Recently, a similar strategy reported that mutant MDV with double deletion of *vIL8* and *meq* resulted in the development of a safe and efficacious MDV vaccine [41]. Thus, it would be intriguing to further study the protection efficacy of LORF9 and *meq* double deletion virus.

### 2.10. MDV071/LORF10

MDV071/LORF10 (Md5, nucleotide: 122,897–122,313) encodes a 194-aa long protein with a predicted MW of 21.4 kDa, and is located at the right terminus of the UL region. MDV071/LORF10 homologues are present in MDV-1 and HVT, but not in MDV-2 genomes [4,19]. MDV MDV071/LORF10 is the homologue of varicella zoster virus (VZV) ORF2, which is dispensable for replication and establishment of latent infection of VZV [42]. We have shown that MDV MDV071/LORF10 is dispensable for virus replication and pathogenesis [36].

### 2.11. MDV072/LORF11

MDV072/LORF11 (Md5, nucleotide: 126,241–123,530) encodes a 903-aa long protein with a predicted MW of 103.2 kDa, and is located at the right terminus of the UL region. MDV072/LORF11 homologues are present in MDV-1, MDV-2, and HVT genomes [4,19]. MDV LORF11 was shown to be dispensable for virus replication in cell culture since deletion of a 2.57 kb fragment of LORF11 from Md5 virus did not have any effect on virus replication in vitro [43]. However, inoculation of chickens with the same mutant virus resulted in significantly lower viremia, compared to the parental virus, and replication in the bursa and FFE was also impaired, suggesting that LORF11 is important for virus replication in vivo [43]. Similarly, LORF11 has also been shown to be important of MDV pathogenesis as infection with LORF11 deletion virus did not cause tumors and mortality in chickens [43].

### 2.12. LORF12

LORF12 homologs of Md5, RB-1B, 648a, and CVI988 encode a 126-aa long protein with a predicted MW of 14.1 kDa; while LORF12 of GA (GA, nucleotide: 125,390–124,944) encodes a 148-aa long protein with a predicted MW of 16.4 kDa (Table 1). LORF12 is unique to MDV-1 and is not present in MDV-2 and HVT. LORF12 is located at the right terminus of the UL region and downstream of MDV073/R-LORF14a (Md5, nucleotide: 127,787–126,915), which encodes phosphoprotein pp38. In a study by Prigge et al., a recombinant virus where a GFP gene was fused to the 3′ end of pp38 gene, and a part of LORF12 was deleted. The resulting recombinant virus was highly attenuated and it was speculated this phenotype could be due to the effect of GFP on normal function of pp38 or because of the partial deletion of LORF12 [44]. Since we have shown that deletion of pp38 coding region only partially attenuated MDV [45], it is reasonable to speculate that the partial deletion of LORF12 contributed, at least partially, to the significant attenuation of the pp38/eGFP recombinant virus.

## 3. MDV US Unique ORFs

Ten ORFs were annotated in the US region of Md5 strain [4,46], including 4 unique ORFs (Figure 1B), and the nucleotide location of each ORF is listed in Table 1. The nucleotide range of their homologues in GA, RB-1B, 648a, and CVI988 are also provided in Table 1. Genes within the US region encode major viral envelop glycoproteins (gD, gI, and gE), and other proteins (such as U_S_3 protein kinase and ICP22) involved in DNA replication and gene expression [47,48,49,50,51,52]. In the last two decades, functional characterization of unique ORFs in US region has uncovered some important functions.

### 3.1. SORF1

SORF1 homologues of GA, RB-1B, 648a, and CVI988 are located in the US region, which encodes protein ranging from 76 to 114-aa long (Table 1). However, its corresponding ORF in Md5 is located entirely within the IRS region (Md5, nucleotide: 153,253–152,981 or 165,191–165,463) and encodes a 90-aa long protein. SORF1 is unique to MDV-1 and is not present in MDV-2 and HVT. Parcells et al. characterized deletion mutant viruses, lacking 4.8 kb or 4.5 kb of the US region [53,54]. The deleted region contains six MDV ORFs, including SORF1, MDV087/SORF2, MDV088/US1, MDV089/US10, MDV090/SORF3, and MDV091/US2. These studies showed that these genes are not essential for virus growth in vitro and dispensable for MDV oncogenicity in chickens [53,54]. However, the specific role of SORF1 in MDV replication and pathogenesis remains to be clarified.

### 3.2. MDV087, MDV097/SORF2

SORF2 homologues of GA, RB-1B, 648a, and CVI988 are located at the left terminus of the US region (Table 1) and encode a 179-aa long protein with a predicted MW of 20.1 kDa. However, its corresponding ORF, MDV087 (Md5, nucleotide: 153,443–153,982), in Md5 genome is located at the IRS/US junction region. Similar to the pattern of genes encoding pp38/pp24, another Md5 ORF, MDV097 (Md5, nucleotide: 165,001–164,552), shares 356 nucleotides with 5′ end sequences of MDV087. SORF2 is unique to MDV-1 and is not present in MDV-2 and HVT. MDV SORF2, a homologue of fowlpox virus (FPV) ORF4 [46], has been shown not to be required for virus replication and oncogenicity [53,54]. In vitro interaction studies showed that SORF2 interacts with chicken growth hormone (GH), which was confirmed by co-immunoprecipitation and co-localization assays [31,55].

### 3.3. MDV090/SORF3

MDV090/SORF3 (Md5, nucleotide: 156,785–155,730) encodes a 351-aa long protein with a predicted MW of 40.1 kDa. MDV090/SORF3 homologues are present in MDV-1, MDV-2, and HVT [4,19]. A deletion MDV mutant that lacks six MDV US ORFs, including SORF1, MDV087/SORF2, MDV088/US1, MDV089/US10, MDV090/SORF3, and MDV091/US2, has been shown to be dispensable for growth of MDV in vitro and MDV oncogenicity in chickens [53,54]. However, the specific role of SORF3 in MDV replication and pathogenesis remains to be studied.

### 3.4. MDV093/SORF4

MDV093/SORF4 (Md5, nucleotide: 159,254–159,697) encodes a 147-aa long protein with a predicted MW of 16.8 kDa, and its importance in MDV pathogenesis remains unknown. MDV093/SORF4 is unique to MDV-1 and is not present in MDV-2 and HVT.

## 4. Summary and Future Direction

In this review, we summarized our current knowledge of unique ORFs encoded within the UL and US regions of MDV. There are also some ORFs in the repeat regions of MDV that remain to be studied. Some of these ORFs partially or completely overlap with other MDV genes; thus, partial or single amino acid mutations, such as those disrupting the start or stop codon, can without affecting the protein sequence of the overlapping gene be used to elucidate specific function of these ORFs.

Other than these genes, taking advantage of RNA sequencing and reverse transcription PCR analyses, some novel ORFs were identified in MDV. For example, two additional splicing variants of gC were identified and have been shown to be required for efficient horizontal transmission of MDV [56]. In addition, several genes within the repeated long region of MDV, including *meq*, RLORF4, and RLORF5a, have been shown to splice with exons II and III of *vIL8*, and the Meq-vIL8 splice variant was demonstrated to be an important virulence factor of MDV [57,58]. Using next generation RNA-sequencing, Bertzbach et al. analyzed the transcriptional landscape of RB-1B and CVI988/Rispens strains of MDV in infected primary chicken B cells and identified novel spliced transcripts, such as SORF6 [59], the role of which in MDV pathogenesis remains to be explored.

## Figures and Tables

**Table 1:** Unique open reading frames (ORFs) encoded by unique long (UL) and short (US) regions of different MDV strains.

Md5 (AF243438.1)		GA (AF147806.2)		RB-1B (EF523390.1)		648a (JQ806361.1)		CVI988 (DQ530348.1)	
ORF	Start–end (aa) ^1^	ORF	Start–end (aa)	ORF	Start–end (aa)	ORF	Start–end (aa)	ORF	Start–end (aa)
MDV009	14,338–13,337 (333)	LORF1	12,894–11,893 (333)	MDV009	15,005–14,004 (333)	LORF1	13,757–12,756 (333)	MDV009	14,786–13,785 (333)
MDV010	14,535–14,630 14,701–16,875 (756)	LORF2	13,091–13,186 13,257–15,431 (756)	MDV010	15,202–15,297 15,368–17,542 (756)	LORF2	13,954–14,049 14,120–16,294 (756)	MDV010	14,983–15,078 15,149–17,323 (765)
MDV011* ^2^	17,431–17,688 (85)	MDV011*	***15,987*–*16,245*** ***(85)***	MDV011*	18,098–18,355 (85)	MDV011*	***16,850–17,107*** ***(85)***	MDV011*	18,136–17,879 (85)
MDV012*	17,828–18,982 (384)	LORF3	16,351–17,547 (398)	MDV012*	18,495–19,649 (384)	LORF3	17,247–18,401 (384)	MDV012*	18,276–19,430 (384)
***LORF4*** ^3^	***19,608*–*19,180 (142)***	LORF4	18,173–17,745 (142)	MDV013.5	20,275–19,847 (142)	***LORF4***	***19,027*–*18,599 (142)***	MDV013.5	20,056–19,628 (142)
***LORF5***	***60,996*–*61,355 (119)***	LORF5	59,557–59,916 (119)	MDV039.5	61,664–62,023 (119)	LORF5	60,415–60,774 (119)	MDV039.5	61,444–61,803 (119)
***LORF6***	***88,119*–*88,586 (155)***	LORF6	86,615–87,082 (155)	MDV049.5	88,832–89,299 (155)	LORF6	87,493–87,960 (155)	MDV049.5	88,510–88,977 (155)
***LORF7***	***91,537*–*91,899 (120)***	LORF7	90,033–90,395 (120)	MDV050.5	92,250–92,612 (120)	LORF7	90,911–91,273 (120)	MDV050.5	91,928–92,290 (120)
***LORF8***	***104,992*–*104,366 (208)***	LORF8	103,493–102,867 (208)	MDV057.8	105,706–105,080 (208)	LORF8	104,366–103,740 (208)	MDV057.8	105,386–104,760 (208)
MDV069	121,289–120,480 (269)	LORF9	119,794–118,985 (269)	MDV069	122,003–121,194 (269)	LORF9	120,665–119,856 (269)	MDV069	121,664–120,855 (269)
MDV071	122,897–122,313 (194)	LORF10	121,399–120,818 (193)	MDV071	123,611–123,027 (194)	LORF10	122,273–121,689 (194)	MDV071	123,272–122,688 (194)
MDV072	126,241–123,530 (903)	LORF11	124,742–122,031 (903)	MDV072	126,955–124,244 (903)	LORF11	125,617–122,906 (903)	MDV072	126,616–123,905 (903)
***LORF12***	***126,823*–*126,443 (126)***	LORF12	125,390–124,944 (148)	MDV072.8	127,537–127,157 (126)	LORF12	126,199–125,819 (126)	MDV072.8	127,198–126,818 (126)
***SORF1***	***153,253*–*152,981 or*** ***165,191*–*165,463 (90)***	SORF1	151,064–150,795 (89)	MDV086.6	154,585–154,241 (114)	SORF1	152,303–152,037 (88)	MDV086.6	154,835–154,605 (76)
MDV087	153,443–153,982 (179)	SORF2	151,254–151,793 (179)	MDV087	154,775–155,314 (179)	***SORF2***	***152,493*–*153,032 (179)***	MDV087	155,025–155,564 (179)
MDV090	156,785–155,730 (351)	SORF3	154,596–153,541 (351)	MDV090	158,117–157,062 (351)	***SORF3***	***155,836*–*154,781 (351)***	MDV090	158,366–157,311 (351)
MDV093	159,254–159,697 (147)	SORF4	157,065–157,508 (147)	MDV093	160,586–161,029 (147)	SORF4	158,305–158,748 (147)	MDV093	160,835–161,278 (147)
MDV097	165,001–164,552 (149)	– ^4^	–	–	–	–	–	–	–

^1^ Start and end nucleotide of ORFs in the genome of different MDV strains, respectively. Length of protein, number of amino acids, is indicated in parenthesis. ^2^ In the most recent Md5 annotation (GenBank: NC_002229.3), MDV011* and MDV012* were annotated as a spliced gene, named MDV012 (Md5, nucleotide: 17,431-17,539, 17,622-18,982), and MDV011* ORF was excluded from the new annotation. ^3^ In case where an ORF is present but has not been annotated, the nomenclature of other strain is used and the predicted nucleotide location is provided in ***bold italics***. ^4^ “–” means ORF not present.

## Data Availability

Not applicable.
